# Fat Men

**Published:** 1857-10

**Authors:** 


*Fat Men.*—The Nashville Med. and Surg. Journal contains an account of a remarkable example of the "adipose diathesis." Tennesee has furnished many men intellectually and morally great. What may be its relative claims in this respect among her sister States, we are not prepared to say, but certain it is that in men of fat she carries off the palm. We quote the account as follows:

"Mr. Darden was born in North Carolina in 1793, and died at his residence in Henderson county, Tennessee, January 23, 1857. He was seven feet six inches high. In 1845 he weighed eight hundred and seventy-one pounds, and at his death a fraction over *one thousand pounds*, and was unquestionably the largest man in the world, and since "those days" in which there were giants, the largest man the world has produced. Up to 1853, "he was quite active and lively, and labored," after which, his fat increasing, he was compelled to stay at home or be hauled about in a two-horse wagon. It required thirteen and a-half yards of flax cloth, a yard wide, to make him a coat. It required sixteen yards of cambric for his shroud, and twenty-four yards of black velvet to cover his coffin."

